# Beneficial Effects of Behavioral Parent Training on Inhibitory Control in Children With Attention-Deficit/Hyperactivity Disorder: A Small-Scale Randomized Controlled Trial

**DOI:** 10.3389/fpsyt.2022.859249

**Published:** 2022-04-27

**Authors:** Akiko Yao, Koji Shimada, Ryoko Kasaba, Akemi Tomoda

**Affiliations:** ^1^Research Center for Child Mental Development, University of Fukui, Fukui, Japan; ^2^Department of Child Development, United Graduate School of Child Development, Osaka University, Kanazawa University, Hamamatsu University School of Medicine, Chiba University, and University of Fukui, Fukui, Japan; ^3^Biomedical Imaging Research Center, University of Fukui, Fukui, Japan; ^4^Department of Child and Adolescent Psychological Medicine, University of Fukui Hospital, Fukui, Japan

**Keywords:** ADHD, behavioral parent training, inhibitory control, response selection, response inhibition

## Abstract

The purpose of this study was to examine whether the beneficial effects of behavioral parent training (BPT), as an indirect type of psychosocial treatment, are extended to cognitive manifestations beyond behavioral symptoms of attention-deficit/hyperactivity disorder (ADHD). Although previous studies of community families have shown an association between parenting quality and a child’s cognitive functions, little is known about the effects of BPT on cognitive manifestations in children with ADHD. In this study, we focused on inhibitory control among cognitive domains, which is considered to be the most malleable to direct types of psychosocial treatment for ADHD. We hypothesized that inhibitory control is affected by BPT, which uses parents as the primary agents of change to help their children. Thirty school-age children (6–12 years old) with ADHD and their parents (mothers) participated and were randomly assigned to either the standard BPT or waitlist control group. Using two objective laboratory-based tasks of inhibitory control (i.e., go/no-go and single response selection tasks), we assessed baseline and post-treatment response inhibition to suppress task-irrelevant responses and response selection to select task-relevant responses. In addition to decreased ADHD symptoms and negative parenting, the BPT group exhibited significantly improved performance in the single response selection task, but not in the go/no-go task, compared with the waitlist control group. Although tentative, these findings partially support our hypothesis that BPT has beneficial effects on the cognitive inhibitory control of ADHD, highlighting the potential for supportive environmental modifications to advance cognitive development in children with ADHD.

## Introduction

Behavioral parent training (BPT) is commonly used as an indirect type of psychosocial treatment for attention-deficit/hyperactivity disorder (ADHD), which is the most common neurodevelopmental disorder in childhood (1, 2). In contrast to child-centered treatment, BPT is indirect because it encourages parents to increase positive parent–child contact and teaches them specific management strategies to cope with children’s behavioral problems (3). In BPT, parents are acknowledged to be the primary agents of change for managing behavioral problems in their children (4). Although there is a discrepancy between parental and independently rated assessments of ADHD symptoms, recent meta-analysis studies (5, 6) have reported that BPT decreases ADHD symptoms, as subjectively reported by parents, who are the most ecologically valid assessors of children’s symptom expression (7).

Given that a transactional model of ADHD and family functioning has suggested that suboptimal parenting practices exacerbate ADHD symptoms and their associated difficulties (8), improved parenting practices via BPT may improve behavioral symptoms and the underlying cognitive mechanisms of ADHD. ADHD’s behavioral symptoms are characterized by deficits in cognitive (neuropsychological) functions, including executive function (EF) implemented mainly in the prefrontal cortex (PFC) (9, 10). Previously, a preventive intervention study of community families demonstrated that a child’s inhibitory control of EF, measured subjectively by parents, improved when supportive and responsive parenting increased (11). Similarly, there are also findings of observational studies showing an association between parenting quality and a child’s cognitive functions by using cross-sectional (12, 13) and longitudinal study designs (14, 15). Here, the current study aimed to investigate the hypothetical effects of BPT on cognitive manifestations in children with ADHD.

To date, improvements in the cognitive domains of ADHD have been examined by several studies using direct types of non-pharmacological psychosocial treatments (16–20). Direct psychosocial treatments target ADHD-related behaviors and cognition with the child as the primary agent of change (e.g., cognitive behavioral therapy, cognitive training, neurofeedback, physical exercise). However, BPT is considered to be an indirect type of psychosocial treatment, as adults are recognized as agents of change (3). As reported by several meta-analyses (16, 18–20), cognitive training is likely to be restricted to near transfer (but not far transfer) effects on cognitive outcomes. A more recent meta-analysis (17) has suggested that of the cognitive domains (e.g., working memory, inhibitory control, flexibility, attention), the inhibitory control component of EF was most affected by a wide range of direct psychosocial treatments and thus could be considered to be the most malleable. Conversely, it is not known whether BPT, as an indirect approach, has beneficial effects on ADHD’s cognitive domains. Although objective cognitive tasks have not been included as outcomes measures in most BPT studies (17), a few BPT studies have examined EF improvements, including inhibitory control and working memory (21–24). However, the results of these studies are limited because they lack control group comparisons. A randomized controlled trial (RCT) using objective cognitive measures is thus needed to produce extended evidence that BPT has beneficial effects on the cognitive domains of children with ADHD.

The current study aims to provide extended RCT evidence of BPT as a current evidence-based standard psychosocial treatment for children with ADHD (4, 25). From the transactional model of ADHD and family functioning as well as the related studies reviewed above, we hypothesized that BPT improves parenting practices and ADHD behavioral symptoms as well as cognitive manifestations in children with ADHD. Of the cognitive functions, we focused on inhibitory control, which is considered to be the most malleable to psychosocial treatments (17). Inhibitory control is assumed to have at least two distinct cognitive operations (26, 27): response inhibition to suppress task-irrelevant information/responses (go/no-go task) and response selection to select task-relevant information/responses (single response selection task). If our hypothesis were supported, the BPT group would exhibit more improvements in behavioral symptoms, inhibitory control, and parenting practices than the waitlist control group.

## Materials and Methods

### Participants

Parents (mothers) and their children aged 6–12 years were recruited in three cohorts through advertisements at the University of Fukui Hospital outpatient clinic. All children were diagnosed with ADHD by child psychiatrists in the outpatient clinic according to the fifth edition of the Diagnostic and Statistical Manual of Mental Disorders (DSM-5) interview (28), based on school reports, observations of the child, and clinical interviews with the family. All children had normal or corrected-to-normal vision. Most (88%) were right handed. All parents had completed at least 12years of education (non-compulsory secondary-level or university-level education) and were thus categorized as having a relatively high education level. Most (84%) were living above the relative poverty line, which was set at 50% of the country’s median household income. Informed consent/assent was obtained from all participants included in this study. The study protocol was approved by the Research Ethics Committee of the University of Fukui and was conducted in accordance with the Declaration of Helsinki and the Ethical Guidelines for Medical and Health Research Involving Human Subjects of Japan.

**FIGURE 1:**
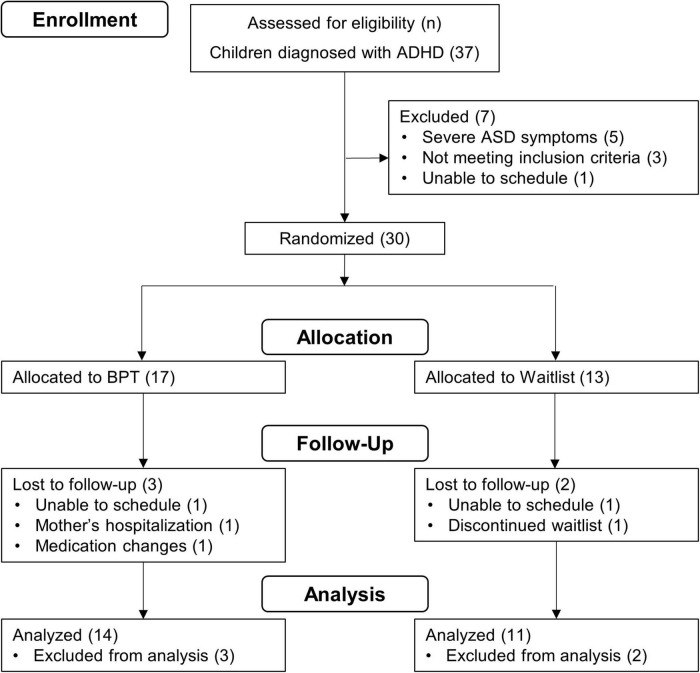
Participant flow diagram.

The inclusion criteria were as follows: Japanese as a first language spoken at home; parenting an elementary school-age child (6–12 years old) with ADHD diagnosis per the DSM-5 (28); and more than mild symptoms of inattention and/or hyperactivity/impulsivity on the Swanson, Nolan, and Pelham rating scale (SNAP) (29, 30). Exclusion criteria were as follows: intellectual disability (IQ < 70) measured by the Wechsler Intelligence Scale for Children (WISC); presence of moderate to severe symptoms of autism spectrum disorder (ASD) per the DSM-5 (28); self-reported psychiatric symptomatology in the parent; and participation in another BPT program within two months of screening. For children taking medication (e.g., osmotic-release oral system formulation of methylphenidate) for ADHD symptoms, parents were asked to maintain their medication status throughout this study. Based on these criteria, 30 eligible participants were selected and randomized into either the BPT or the waitlist control group (Figure 1).

### Procedure

Participants were randomly assigned to either the BPT (*n* = 17) or the waitlist control (*n* = 13) group using a permuted-block randomization with varying block/cohort size, after sufficient participants meeting the criteria were recruited. Randomization was conducted by an independent research coordinator using a computer-generated random number sequence. The time between baseline (T1) and post-treatment (T2) assessments for the two groups was approximately 13 weeks. The BPT group participated in a 13-week BPT program (see section “Behavioral Parent Training”) between T1 and T2, whereas the waitlist control group received no training. Additionally, they did not have any form of social contact with the researchers between T1 and T2. Because of ethical considerations, participants in the waitlist control group were promised the same BPT program in the future. Both groups were assessed using behavioral and cognitive (neuropsychological) measures at baseline and post-treatment. Parents answered questionnaires to assess children’s ADHD symptoms and related behavioral problems, and parents’ parenting practices and mental health. Children performed laboratory cognitive measures of inhibitory control (go/no-go and single response selection tasks) and working memory storage (digit span task).

### Behavioral Parent Training

Behavioral parent training (BPT) was conducted in person in a group format across 13 consecutive, 2-h weekly sessions for each cohort. Each group session was facilitated by two behaviorally trained female clinical psychologists. To maintain treatment fidelity, the psychologists covered the BPT content by conducting sessions with the BPT program guidebook in all sessions (31). They reviewed the topics to be covered before each session and checked the coverage after the session. Any topic omitted from a session was presented in the following session. The BPT was based on a culturally adapted version of the BPT program (31), which originated from the UCLA and Barkley BPT programs for families of children with a wide range of disorders/disabilities, including ADHD (32, 33). The BPT content aimed to increase positive parent–child interactions and parental communication skills, while reducing parent–child conflicts and child oppositional defiant behaviors through lectures, group discussions, modeling, and role playing. Weekly homework assignments facilitated BPT skill implementation at home.

There were 13 weekly sessions of the BPT program, and each session lasted approximately 2 h (31–33). Psychological education for ADHD and parent’s stress managements were taught in sessions 1–3. Parenting skills for observing the child’s behaviors and paying positive attention to the child’s adaptive behaviors were taught in sessions 4–6. Parenting skills for providing clear rule, clear instruction and more structure in time and space were taught in sessions 7–9. Techniques for planned ignoring of child’s non-adaptive behaviors were taught in sessions 10–12. Session 13 was a wrap-up. Parents were asked to complete homework, specific to each session.

### Subjective Questionnaire Measures

The Parenting Scale (PS) (34, 35) was used to assess parenting style/discipline practices, including laxness and over-reactivity. Parents rated their discipline strategies such as spanking and yelling, as forms of harsh physical and verbal discipline, on a seven-point Likert scale. The spanking ratings ranged from “*I spank, slap, grab, or hit my child”* (7) to “*Never or rarely*” (1) in the discipline situation “*when my child misbehaves*,” and the yelling ratings ranged from “*I raise my voice or yell”* (7) to “*I speak to my child calmly*” (1). The Parenting Stress Index (PSI) (36, 37) evaluated parenting stress (child and parent domains).

Attention-deficit/hyperactivity disorder (ADHD) and oppositional defiant disorder (ODD) symptoms were assessed using parent-reported ratings on the SNAP (29, 30). Parents rated their children’s inattentive (items 1–9), hyperactive/impulsive (items 10–18), and defiant (items 19–26) behaviors using a four-point Likert scale (0 = not at all, 1 = just a little, 2 = quite a bit, 3 = very much). Higher scores in the SNAP indicates more severe ADHD and ODD symptoms. The subscale scores of > 9.0 for inattention and hyperactivity/impulsivity are clinically suggestive of ADHD (30, 38). To assess children’s problems with regulating affect, behavior, and cognition, parents rated the Child Behavior Checklist (CBCL/6-18) (39, 40), which included the Anxious/Depressed, Aggressive Behavior, and Attention Problems scales (41).

### Objective Cognitive Measures

Response inhibition and selection were assessed by two objective, computer-based tasks of inhibitory control (go/no-go and single response selection tasks) (26, 27, 42). In each task, the children were required to discriminate a target stimulus from non-target stimuli. Each child determined the target stimulus from five characters of a popular video game (*Super Mario Brothers*) to facilitate motivation for task engagement. In the go/no-go task, participants were instructed to press a response key on a computer keyboard with the right hand as soon as the target (go) character stimulus (e.g., Mario) appeared in the center of the screen, but to withhold the response if one of the non-target (no-go) character stimuli (e.g., Luigi, Princess Peach) appeared. The stimulus duration was 500 ms and the interstimulus interval was randomly varied between 1100 and 1900 ms. After short practice trials, participants performed 100 trials of the task (80 go and 20 no-go trials). Commission error (CE) reflected failed inhibitions (i.e., incorrect go response to no-go trials) as the primary index of the task. The secondary indexes were omission error (OE), response time (RT), and RT variability (RTV).

In the single response selection task, participants were instructed to press one of two response keys on a computer keyboard with the right hand as soon as the target character stimulus (e.g., Mario) appeared in the center of the screen and to press a different key with the left hand if one of the non-target object stimuli (e.g., Coin, Flower) appeared. The stimulus duration was 500 ms and the interstimulus interval was randomly varied between 1500 and 2400 ms. After short practice trials, participants performed 80 trials of the task (10 target and 70 non-target selection trials). RT to target trials reflected fluent selection as the primary index of the task. The secondary indexes were target trial errors (i.e., incorrect non-target responses to target trials) and non-target trial errors (i.e., incorrect target response to non-target trials).

In addition, the forward digit span task was used to assess working memory storage. A sequence of digits appeared in the center of the screen at a rate of one digit every 1000 ms, and participants were instructed to immediately recall each sequence in the same order. The task began with a sequence length of two digits and increased by one digit after two trials. The task ended when the recalled sequence was incorrect in two trials of the same length. The longest correctly recalled sequences were used as the key measure of working memory storage.

### Data Analysis

All analyses were based on per-protocol analysis where participants who received the BPT program or the waitlist schedule were included. Participants who dropped out of the BPT program or the waitlist schedule were excluded from the analyses. Owing to the small-scale nature of the trial, we decided that intention-to-treat analysis was not viable and could be misleading (43). The BPT and waitlist control groups were compared on all outcome measures at baseline (T1) and post-treatment (T2). For the questionnaire measures, missing item responses were imputed using the individual mean imputation method, which imputes the calculated mean of a given participant’s complete responses to other items of the same scale. The missing response rates were 0.13% in the BPT group and 0.08% in the waitlist control group, which were not significantly different (*p* = 0.555). For the cognitive measures, because of technical problems, data from two participants in the go/no-go task and five participants in the single response selection task were not recorded, and were not included in data analyses. A two-way analysis of variance (ANOVA) was conducted with group (BPT, waitlist control) as a between-subjects factor and time (T1, T2) as a within-subjects factor. An interaction between these two factors indicated a change in the treatment effects between T1 and T2. Effect size (Cohen’s *d*) calculations were based on Morris’s formula (44), where the mean baseline to post-treatment change in the BPT group minus the mean baseline to post-treatment change in the waitlist control group was divided by the pooled baseline standard deviation. Statistical analyses were conducted using IBM SPSS Statistics version 26 (IBM Japan, Tokyo, Japan). All *p*-values below 5% were considered to be statistically significant.

## Results

### Allocation, Dropout, and Engagement

Thirty participants were randomized (Figure 1). Seventeen were allocated to the BPT and 14 of these received the treatment program from T1 to T2. Participants attended most of the BPT program sessions (88.17% of all 13 sessions), indicating higher attendance rates (fidelity of treatment receipt) relative to the 71% in previous group-format BPT studies (45). For the waitlist control group, 13 participants were allocated and 11 of these received a waiting schedule from T1 to T2. Post-treatment (T2) data were obtained for 14 participants in the BPT group and 11 in the waitlist control group.

### Baseline Characteristics

Table 1 presents the demographic characteristics of the parents and children in the BPT and waitlist control groups. There were no significant between-group differences for any assessed parent (age, education, relative poverty) or child (age, sex, handedness, IQ, medication status, ASD diagnosis, ADHD symptoms) characteristics (*ps* > 0.102). For subjective questionnaire outcomes at baseline (Table 2), the BPT and waitlist control groups were equivalent in terms of the PS, PSI, SNAP, and CBCL (*ps* > 0.299). Furthermore, for objective cognitive outcomes (Table 3), these two groups did not significantly differ on any baseline measures (*ps* > 0.077).

**TABLE 1:** Participant characteristics.

	BPT (*n* = 14)			Waitlist (*n* = 11)			χ*^2^*	*t*
	M	SD	%	M	SD	*%*		
**Parents (Mothers)**								
Age	38.21	(5.01)		40.18	(4.38)			−1.03
Education (≥12 years)			100			100		
Living above the relative poverty line			92.9			72.7	1.86	
**Children**								
Age	8.96	(1.65)		9.70	(1.82)			−1.06
Sex (female)			21.4			0	2.68	
Right-handed			92.9			81.8	0.71	
Intelligence quotient (WISC)	99.50	(13.38)		98.18	(13.57)			0.24
Medication			64.3			72.7	0.20	
ASD diagnosis			28.6			45.5	0.76	
**ADHD symptoms (SNAP)**								
Inattention	16.79	(4.78)		14.82	(4.36)			1.06
Hyperactivity/impulsivity	10.79	(5.28)		8.91	(6.02)			0.83
Opposition/defiance	7.86	(4.04)		8.73	(6.84)			−0.40

*ADHD, attention-deficit/hyperactivity disorder; ASD, autism spectrum disorder; SNAP, Swanson, Nolan, and Pelham rating scale, WISC, Wechsler Intelligence Scales for Children.*

**TABLE 2:** Subjective (parent-reported) questionnaire outcomes for the BPT and waitlist control groups.

	BPT				Waitlist				*F*	ES (*d*)
	T1		T2		T1		T2			
***PS [n* = 14/11 *(BPT/Waitlist)]***										
Yelling	5.14	(1.41)	3.21	(1.42)	5.36	(1.29)	5.27	(1.42)	12.01**	−1.31
Spanking	3.93	(2.17)	2.57	(1.45)	4.45	(1.44)	4.00	(1.73)	2.41	−0.47
Over-reactivity	40.93	(11.17)	34.29	(8.19)	44.44	(8.98)	43.64	(11.66)	3.21^†^	−0.55
Laxness	22.50	(6.09)	22.55	(7.41)	21.91	(5.91)	22.00	(6.56)	0.00	−0.01
***PSI (n* = 14/11**)										
Child domain	106.86	(11.02)	100.61	(12.02)	107.18	(15.36)	111.27	(16.99)	5.34*	−0.76
Parent domain	108.43	(22.36)	104.29	(20.48)	103.00	(19.94)	112.64	(14.29)	7.94*	−0.72
***SNAP (n* = 14/11**)										
Inattention	16.79	(4.78)	16.64	(3.84)	14.82	(4.36)	18.45	(3.88)	7.07*	−0.80
Hyperactivity/impulsivity	10.79	(5.28)	10.00	(4.54)	8.91	(6.02)	10.27	(5.61)	3.83^†^	−0.37
Opposition/defiance	7.86	(4.04)	8.21	(4.85)	8.73	(6.84)	10.36	(7.28)	0.62	−0.23
***CBCL (n* = 14/11**)										
Anxious/depressed	4.50	(2.65)	4.21	(2.86)	5.73	(4.78)	6.39	(5.17)	0.79	−0.25
Attention problems	10.64	(2.76)	9.50	(2.07)	10.55	(3.24)	11.45	(3.08)	3.38^†^	−0.66
Aggressive behavior	11.07	(4.83)	10.44	(5.89)	11.00	(7.40)	13.27	(8.20)	3.41^†^	−0.46

Mean (SD); BPT, behavioral parent training; CBCL, Child Behavior Checklist; ES, effect size (Cohen’s d); PS, Parenting Scale; PSI, Parenting Stress Index; SNAP, Swanson, Nolan, and Pelham rating scale; T1, baseline; T2, post-treatment.^†^p < 0.1; *p < 0.05; **p < 0.01.

**TABLE 3:** Objective cognitive task outcomes for the BPT and waitlist control groups.

	BPT				Waitlist				*F*	ES (*d*)
	T1		T2		T1		T2			
***Go/no-go task [n* = 13/10 *(BPT/Waitlist)]***										
Commission error (%)	33.46	(25.12)	33.08	(29.83)	35.50	(30.04)	35.00	(30.09)	0.00	0.00
Omission error (%)	2.88	(4.49)	1.73	(2.37)	1.38	(1.24)	1.25	(2.04)	0.37	−0.28
RT (ms)	373.23	(84.99)	389.53	(81.29)	339.42	(60.11)	344.21	(59.20)	0.14	0.15
RT variability (ms)	126.47	(69.37)	137.51	(67.22)	88.38	(23.01)	104.63	(32.25)	0.06	−0.09
***Single response selection task (n* = 10/10)**										
RT to target trials (ms)	519.03	(117.45)	473.64	(117.72)	441.13	(92.88)	517.70	(130.97)	4.45*	−1.10
Error of target trials (%)	34.00	(19.55)	46.00	(20.11)	42.00	(26.16)	37.00	(24.97)	2.71	0.71
Error of non-target trials (%)	7.27	(9.21)	3.93	(3.29)	8.67	(6.96)	6.93	(5.55)	0.16	−0.19
***Forward digit span task (n* = 14/11)**										
Recalled sequence	4.50	(0.76)	5.14	(1.17)	5.09	(0.83)	5.45	(1.44)	0.56	0.34

Mean (SD); BPT, behavioral parent training; ES, effect size (Cohen’s d); T1, baseline; T2, post-treatment. *p < 0.05.

### Subjective Questionnaire Outcomes

Table 2 presents the descriptive data and effect sizes of the questionnaire outcomes. There were significant interactions between group (BPT, waitlist control) and time (T1, T2) on four measures: yelling discipline rating [*F*(1,23) = 12.01, *p* = 0.002, *d* = −1.31], PSI child domain score [*F*(1,23) = 5.33, *p* = 0.030, *d* = −0.76], PSI parent domain score [*F*(1,23) = 7.94, *p* = 0.010, *d* = −0.72], and SNAP Inattention score [*F*(1,23) = 7.07, *p* = 0.014, *d* = −0.80]. These interactions showed that the BPT group had significant reductions from T1 to T2 in the questionnaire measures compared with the waitlist control group. The remaining scores showed no significant interactions (*ps* > 0.063).

### Objective Cognitive Outcomes

Table 3 presents the descriptive data and effect sizes for the cognitive outcomes. For the go/no-go task, there were no significant interactions between group and time on the primary (CE) and secondary indexes (OE, RT, RTV) (*ps* > 0.548). For the single response selection task, significant interactions were found in the primary index, i.e., the RT to target trials [*F*(1, 18) = 4.45, *p* = 0.049, *d* = −1.10], but not in the secondary indexes, i.e., target trial and non-target trial errors (*ps* > 0.117). The BPT group exhibited significant reductions from T1 to T2 on the response selection task index compared with the waitlist control group (-45.39 ms vs. 76.56 ms). Furthermore, the forward digit span task showed no significant interactions in the recalled sequence (*p* = 0.462).

## Discussion

The current study examined whether BPT effects extend to cognitive manifestations beyond the behavioral symptoms in children with ADHD. The objective cognitive measures from our small-scale RCT study showed that compared with the waitlist control group, the BPT group performed better in the single response selection task (response selection), but not in the go/no-go (response inhibition) or digit span tasks (working memory storage), partially supporting the hypothetical effects of BPT on the cognitive manifestations of ADHD. Moreover, in line with recent meta-analyses (5, 6), the BPT group showed decreased ADHD symptoms and decreased negative parenting and parental stress based on parent-reported assessments. From a view of the transactional model of ADHD and family functioning that suboptimal parenting practices exacerbate ADHD symptoms and their associated difficulties (8), improved parenting practices in the BPT group may improve behavioral symptoms and cognitive manifestations of ADHD and parental stress. However, suboptimal parenting practices in the waitlist control group are likely to negatively influence these outcomes.

To the best of our knowledge, this small-scale RCT study is the first to investigate the effects of BPT on inhibitory control, including response selection and inhibition, in children with ADHD, although previous ADHD studies have examined changes in response inhibition through BPT without employing a control group comparison (22). The BPT group in the current study displayed a more fluent response selection to specific external stimuli (or the mapping of sensory input to a motor response) than the waitlist control group. In the BPT program, parents learned to scaffold (support) their child through routines across the day and other cue-based reminders (e.g., lists of tasks to be completed), and feedback and contingencies to reinforce the successful implementation of daily activities and tasks (31, 32). The parents are encouraged via the BPT program to reward alternative adaptive behaviors to the non-adaptive (unwanted) behaviors in their child. Through such parental scaffolding, the child is supported in making effective selection between the non-adaptive and adaptive behaviors in real-world (information-rich) situations, rather than simply suppressing the non-adaptive behaviors. Consistent with the BPT program’s content and practice, the child’s hyperactive/impulsive behaviors did not decrease statistically but the inattentive behaviors decreased, which may be dependent on different inhibitory control components (response inhibition and selection) of EF. Correspondingly, children’s EF can be improved through parental scaffolding (supporting) (46). As reported by a recent meta-analysis study (17), in addition to this indirect type of psychosocial treatment (i.e., BPT) for children with ADHD, their EF, specifically inhibitory control, was affected by a wide range of direct psychosocial treatments. Regarding cognitive training, the effects on cognitive outcomes were significantly beneficial but were restricted to near transfer cognitive functions (16, 18–20). In particular, significant, small magnitude effects were evident among the 11 studies that included objective cognitive performance, such as inhibitory control tasks (20). For example, a previous RCT study using cognitive training found that treatment group children with ADHD exhibited faster performance in response selection, measured by a Stroop interference task, than the comparison group (47). The direction of RT changes for the task across treatment was consistent with that of developmental trajectories in inhibitory control (48), which indicated faster performance with age (6 to 18 years of age) despite high and low symptoms of ADHD.

Previous studies have suggested that inhibitory control is more malleable to psychosocial ADHD treatments than other cognitive functions such as basic working memory (17). However, it is important to understand why BPT’s beneficial effects in this study were found only for response selection, but not for response inhibition, even though these two cognitive operations are included within inhibitory control (26, 27). A possible reason for this may be associated with the predominantly right-lateralized, particularly PFC deficits in ADHD (49–51), suggesting that the right hemisphere PFC functions in ADHD have less potential for malleability to experience and treatment than the left hemisphere PFC functions. There is evidence of a partially dissociated lateralization of response selection and inhibition in inhibitory control (26, 42, 52–54): the left hemisphere PFC is predominantly involved in response selection, while the right hemisphere PFC is responsible for response inhibition. Regarding inhibitory control in ADHD, as summarized by meta-analysis studies of neuroimaging (50, 55), the right PFC activation during response inhibition (e.g., go/no-go task) is prominently reduced in children with ADHD compared with those without ADHD, supporting the theory of right-lateralized deficits in ADHD (51). Such hypoactivation in the right PFC during response inhibition in ADHD is known to be increased through direct psychosocial treatment (i.e., neurofeedback training) for children with ADHD (56). More direct treatments of the underlying neurocognitive causes of ADHD may be needed to improve the response inhibition anchored in the right PFC. Combined with the most prominent delayed maturation of the right PFC surface area in ADHD (57), our findings on the different effects of BPT on inhibitory control in children with ADHD may result from the different potential for malleability (plasticity) in the left and right PFC lateralization of response selection and inhibition. Further studies with functional neuroimaging techniques are necessary to better elucidate the neural mechanisms for the different effects of BPT on different inhibitory control components.

Although children’s cognitive functions (e.g., EF) have not previously been examined in most BPT studies (17), it is not surprising that BPT effects were found to be partially associated with cognitive manifestations beyond behavioral symptoms in children with ADHD. Parental negative discipline (e.g., verbal punishment) in response to a child’s misbehavior was decreased via BPT in our study, which may contribute a more supportive home environment that improves children’s EF (e.g., response selection). This is consistent with the traditional view (58) that children’s cognitive skills are socially constructed through interactions with supportive, responsive adults. A transactional model of ADHD and family functioning has also suggested that suboptimal parenting practices exacerbate children’s ADHD symptoms and their associated difficulties (8). Previous studies including a community sample have found an association between parenting behaviors and children’s EF using cross-sectional (12, 13) and longitudinal observational study designs (14, 15). There is also evidence that negative parenting (e.g., hostility) may have a larger impact on children’s EF than positive parenting (e.g., warmth) (59). Executive function is likely to be influenced by a wide range of circumstances and experiences, specifically during periods of relative plasticity in EF-related neural systems (e.g., PFC), including the preschool years, transition to adolescence, and late adolescence (46). Furthermore, improved parenting behaviors (e.g., using praise) via BPT may enhance children’s motivation by reinforcing their efforts in changing their own behaviors at home (60), which may then affect EF. Regarding the relationship between motivation and cognitive control (EF), a recent framework (61, 62) has argued that higher motivation can offset the higher cognitive control costs in shaping goal-directed selection and behaviors that are likely implemented by a motivationally triggered dopamine release in the PFC. Thus, BPT’s effects on children’s EF are also likely to be associated with their motivation, which is enhanced by improved parenting behaviors via BPT.

Finally, the limitations of the current study should be taken into consideration in future studies. First, given the relatively small sample size, this study was slightly underpowered. Thus, other potentially significant findings may have been neglected. The effects of BPT on other subjective (i.e., PS over-reactivity, CBCL attention problems) and objective measures (i.e., error of target trials in single response selection task) did not reach statistical significance, but showed medium to large effect sizes. The small sample size may diminish the statistical power of the effects. Studies involving a larger number of participants are essential to replicate and generalize our results. Second, most children in this study were taking concurrent medication treatment, which may limit the generalizability of our results. However, there were no differences for medication status between the BPT and waitlist control groups. Third, for generalization of our results, the participant’s characteristics (e.g., socioeconomic status, parental education) and the treatment properties (e.g., content, practice) may need to be taken into consideration. For example, it has been previously reported that the participants with lower socioeconomic status profited less from the treatment (63). Moreover, the treatment is more likely to be effective when the content most closely approximates the behavior targeted in daily life and the duration of practice and feedback is high (25). Fourth, regarding the subjective questionnaire outcomes, the beneficial effects of BPT may be overestimated due to treatment expectancy biases of unmasked parents (5), although they are the most ecologically valid assessors of children’s symptom expression (7). Further studies with independent masked assessments are required to avoid an overestimation of the beneficial effects of BPT on subjective outcomes. Fifth, the objective cognitive measures used in this study were limited to one EF component (i.e., inhibitory control) in children with ADHD. Three partially separable EF components are assumed to be inhibitory control, working memory updating, and set shifting (flexibility) (46). Although inhibitory control—the focus of this study—is more malleable to psychosocial treatments for ADHD than other cognitive functions (17), neurocognitive heterogeneity has been increasingly recognized as a valid ADHD phenomenon (10, 64–66). In addition to assessing inhibitory control (EF) and working memory storage (non-EF), further studies assessing multiple neurocognitive constructs (e.g., a hot-cool EF continuum) with multiple tasks would help better understand ADHD’s neurocognitive heterogeneity, allowing us to tailor psychosocial ADHD treatments according to neurocognitive subtypes.

## Conclusion

In this study, we found that in addition to decreased ADHD symptoms and decreased negative parenting, the BPT group exhibited significantly improved performance in the single response selection task, but not in the go/no-go or digit span tasks, compared with the waitlist control group. Improvements in cognitive inhibitory control have previously been demonstrated by direct, child-centered psychosocial ADHD treatments (17). Although tentative, the current study provides partial evidence that BPT, as an indirect type of psychosocial treatment, has beneficial effects on cognitive inhibitory control (specifically response selection) beyond ADHD’s behavioral symptoms, highlighting the potential for supportive environmental modifications for cognitive development in children with ADHD.

## Data Availability Statement

The raw data supporting the conclusions of this article will be made available by the authors, without undue reservation.

## Ethics Statement

The studies involving human participants were reviewed and approved by the Research Ethics Committee of the University of Fukui, Japan. Written informed consent to participate in this study was provided by the participants’ legal guardian/next of kin.

## Author Contributions

AY, KS, RK, and AT conceptualized and designed the study. AY, KS, and RK collected and analyzed the data. AY and KS wrote the first draft of the manuscript. All authors edited and revised subsequent drafts of the manuscript, approved the final manuscript as submitted, and agreed to be accountable for all aspects of the work.

## Conflict of Interest

The authors declare that the research was conducted in the absence of any commercial or financial relationships that could be construed as a potential conflict of interest.

## Publisher’s Note

All claims expressed in this article are solely those of the authors and do not necessarily represent those of their affiliated organizations, or those of the publisher, the editors and the reviewers. Any product that may be evaluated in this article, or claim that may be made by its manufacturer, is not guaranteed or endorsed by the publisher.
